# Biofilm-Forming Lactic Acid Bacteria in Sausages: Isolation, Characterization, and Inhibition Using *Eisenia bicyclis*-Based Nanoparticles

**DOI:** 10.3390/antibiotics14070637

**Published:** 2025-06-22

**Authors:** Do Kyung Oh, Du-Min Jo, Minji Kim, Jeong-Bin Jo, Ji-Hwan Choi, Jeong Mi Choi, Geum-Jae Jeong, Se Yun Jeong, Fazlurrahman Khan, Young-Mog Kim

**Affiliations:** 1 Department of Food Science and Technology, Pukyong National University, Busan 48513, Republic of Korea; 2 Marine Integrated Biomedical Technology Center, The National Key Research Institutes in Universities, Pukyong National University, Busan 48513, Republic of Korea; 3 Research Center for Marine Integrated Bionics Technology, Pukyong National University, Busan 48513, Republic of Korea; 4 National Marine Biodiversity of Korea (MABIK), Seochun 33662, Republic of Korea; 5 Solution Center, Dynesoze Co., Ltd., R&D Center, Yongin 16827, Republic of Korea; 6 Ocean and Fisheries Development International Cooperation Institute, Pukyong National University, Busan 48513, Republic of Korea; 7 International Graduate Program of Fisheries Science, Pukyong National University, Busan 48513, Republic of Korea

**Keywords:** in situ biofilm inhibition, lactic acid bacteria, *Eisenia bicyclis*, phloroglucinol, gold nanoparticle

## Abstract

**Background/Objectives:** Lactic acid bacteria produce biofilms in meat products that contribute to the products’ deterioration, reduction in quality, and shortened shelf life. Although LAB are generally considered benign, certain strains create slime and cause significant drops in pH. The study’s goal was to identify and characterize LAB strains from sausage products that are capable of biofilm formation, and to evaluate the inhibitory effects of *E. bicyclis* methanol extract, its ethyl acetate fraction, and phloroglucinol, as well as to synthesize AuNPs, and assess their efficacy in controlling biofilm formation. **Methods:** Slime or biofilm-producing LAB bacteria were isolated from commercial sausages and identified using 16S rRNA gene sequencing. *Lactobacillus sakei* S10, which can tolerate high salt concentrations and cold temperatures, was chosen as a representative strain. The isolates were subsequently tested for hemolytic activity, salt and temperature tolerance, and carbohydrate consumption patterns. To evaluate antibiofilm potential, marine-derived compounds from *Eisenia bicyclis*, such as phloroglucinol (PG), crude methanolic extracts, ethyl acetate fractions, and gold nanoparticle (AuNP) formulations, were tested in situ on sausage surfaces against *L. sakei* S10 and common pathogens (*Pseudomonas aeruginosa* and *Staphylococcus aureus*). The biofilm-inhibitory effects of the extracts, PG, and PG-AuNPs were estimated using the colony-counting method. **Results:** The PG-AuNPs had an average particle size of 98.74 nm and a zeta potential of −29.82 mV, indicating nanoscale dimensions and considerable colloidal stability. Structural analysis confirmed their spherical form and crystalline structure, as well as the presence of phenolic groups in both reduction and stabilization processes. Among the studied treatments, the PG and PG-AuNPs had the strongest antibiofilm activities, dramatically lowering biofilm biomass, particularly for *P. aeruginosa* and *L. sakei* S10. However, the inhibitory effects were less prominent in in situ conditions than in in vitro testing, highlighting the complexity of real food matrices. **Conclusions:** The results of this study indicate that polyphenolic compounds obtained from marine sources, particularly in nano-formulated forms, have a great deal of potential as natural antibiofilm products. Enhancing the microbiological safety of processed meat products and extending their shelf life could be accomplished through the application of these polyphenolic compounds in food packaging or surface treatments.

## 1. Introduction

Lactic acid bacteria (LAB) are widely recognized as beneficial microorganisms owing to their essential roles in food fermentation and their probiotic effects, which promote gut health and immune function. They are extensively employed in the production of dairy products, fermented vegetables, and processed meats, where they enhance sensory attributes and contribute to microbial safety [1]. Additionally, numerous LAB strains synthesize antimicrobial agents, including organic acids and bacteriocins, which effectively suppress the growth of pathogenic and spoilage microorganisms [2]. These characteristics have established LAB as valuable agents in the food industry, functioning both as starter cultures and natural preservatives. Among the lactic acid bacteria commonly associated with fermented foods, *Lactobacillus sakei* has recently come under the spotlight, as some of its strains have been found to exhibit promising probiotic characteristics [3].

However, the role of LAB in food systems is not exclusively beneficial. Under specific conditions—particularly in refrigerated or vacuum-packed processed meat products—certain LAB strains may act as spoilage organisms rather than protective agents [4,5]. Species such as *Weissella viridescens*, *Latilactobacillus sakei*, and *Leuconostoc* spp. have been linked to spoilage, including slime formation, gas production, sour odor, pH reduction, and undesirable alterations in texture and color [6,7,8]. These changes degrade the sensory quality and shorten the shelf life of meat products, leading to economic losses and decreased consumer acceptance. Moreover, the ability of LAB to form biofilms during post-processing and distribution poses additional concerns [9,10]. These biofilms—structured communities of microbial cells embedded within a self-produced extracellular matrix—can adhere to food surfaces and processing equipment, serving as persistent contamination sources and exhibiting resistance to standard cleaning and sanitation protocols [11,12].

In addition to LAB, pathogenic bacteria such as *Pseudomonas aeruginosa* and *Staphylococcus aureus* pose significant hazards in meat processing environments. These pathogens are capable of forming robust biofilms, which enhance their resilience under stress conditions and elevate the risk of foodborne illness [13,14]. Although chemical preservatives, such as sorbic acid, are legally permitted and widely used to control microbial growth in processed meats—up to 2.0 g/kg according to the Korean Food Code—such agents are increasingly scrutinized due to potential health concerns and rising consumer demand for clean-label products [15]. Furthermore, microbial contamination that occurs during post-processing distribution often falls beyond the protective scope of conventional preservatives, underscoring the limitations of current food safety strategies [15,16].

Consequently, recent research has increasingly focused on developing alternative preservation strategies that are both effective and aligned with consumer preferences. One promising approach involves the use of bioactive packaging materials designed to actively inhibit microbial growth and prevent biofilm formation. In this context, natural extracts derived from plant and marine sources have attracted significant interest due to their antimicrobial and antioxidant properties [17]. While terrestrial plants have traditionally served as the primary source of natural preservatives, marine-derived compounds represent an underexplored reservoir of bioactive substances with distinctive functional attributes [18,19]. Among these, *Eisenia bicyclis*, a brown macroalga rich in phlorotannins, has demonstrated notable antimicrobial and antibiofilm potential [20,21].

Despite their promising bioactivity, the practical application of natural extracts in food systems is often hindered by challenges such as poor solubility, low stability, and limited bioavailability. To address these limitations, nanotechnology-based delivery systems have been proposed. Nanoparticles (NPs) can improve the stability and controlled release of bioactive compounds, while also enhancing their penetration and efficacy at target sites [22]. Among various nanomaterials, gold nanoparticles (AuNPs) have garnered considerable attention due to their excellent biocompatibility, low cytotoxicity, and multifunctional properties, including antibacterial, antioxidant, and antibiofilm activities [23]. Although AuNPs have diverse applications in biomedicine, their safety is a concern, where reports showed that AuNP toxicity is size-dependent, with smaller particles generally being more toxic [24,25,26]. However, some research indicates that certain size ranges (8–37 nm) may be more harmful than others [25]. However, surface functionalization can affect toxicity, with cationic AuNPs being more toxic than anionic ones [27]. When synthesized using natural extracts, AuNPs retain the functional properties of the conjugated compounds, exhibit enhanced antimicrobial performance through synergistic effects, and have excellent biocompatibility [28,29].

Building on these findings, the present study aimed to isolate and characterize LAB strains from sausage products that are capable of biofilm formation, and to assess the inhibitory effects of *E. bicyclis* methanol extract (EB), its ethyl acetate fraction (EA), and phloroglucinol (PG). Additionally, AuNPs were synthesized using each extract—resulting in EB-AuNPs, EA-AuNPs, and PG-AuNPs—and evaluated for their efficacy in controlling biofilm formation by both LAB and pathogenic bacteria under in situ conditions. Through these investigations, we aimed to explore the feasibility of incorporating extract-functionalized AuNPs into active packaging systems as a natural and sustainable approach to improve the microbial safety and quality of processed meat products.

## 2. Results and Discussion

### 2.1. Identification of the Biofilm-Forming LAB Isolated from Meat Products

**Figure 1:**
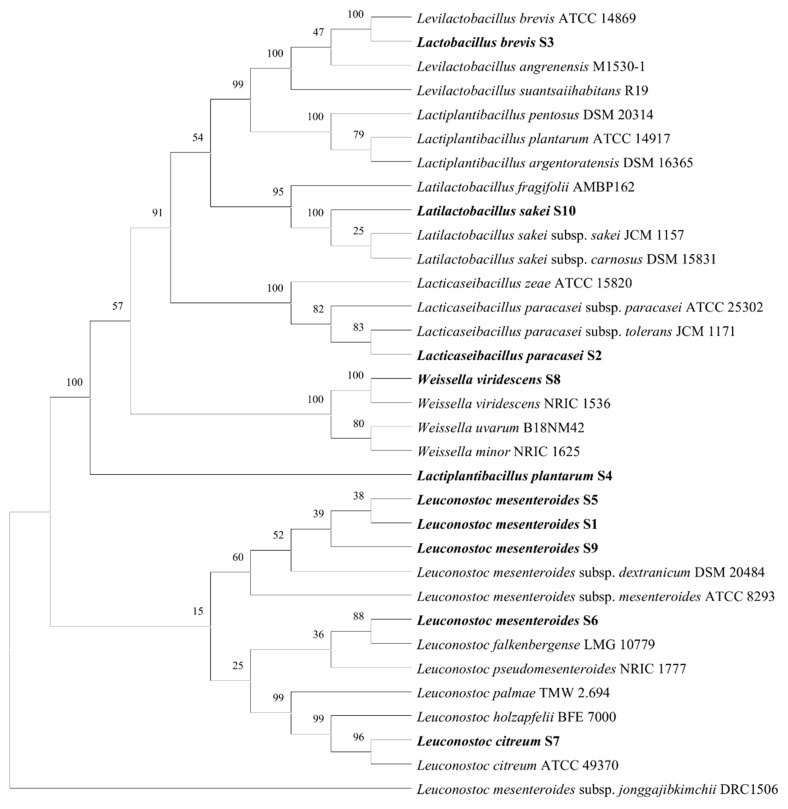
Phylogenetic tree analysis of the LAB isolated from meat products. Bold strains indicate isolates characterized in this study.

Figure 1 displays the results of the identification process completed on ten different LAB strains that were isolated from meat products. Following the classification of the isolates according to the kind of meat product, a phylogenetic tree was created in order to highlight the genetic links that exist between the isolates. The identities of the isolates were established by sequencing the 16S rRNA gene, which was then followed by a BLAST analysis utilizing the NCBI database (https://blast.ncbi.nlm.nih.gov/Blast.cgi, accessed on 18 June 2025). The bacteria that were isolated were identified as follows: *Lacticaseibacillus paracasei* (*n* = 1), *Leuconostoc citreum* (*n* = 1), *Leuconostoc mesenteroides* (*n* = 4), *Levilactobacillus brevis* (*n* = 1), *Lactiplantibacillus plantarum* (*n* = 1), *L. sakei* (*n* = 1), and *Weissella viridescens* (*n* = 1). An investigation of the evolutionary connections between the isolates and the reference strains was carried out by means of a phylogenetic tree that was constructed on the basis of matched 16S rRNA gene sequences [30]. The isolates were given the designations S1 through S10 so that they would be consistent in subsequent studies.

Recent research has shown that certain strains of *L. plantarum, L. mesenteroides*, and *L. sakei* are capable of creating slime layers and biofilms within the sausage matrix [16,31]. Some strains of *L. sakei* have additionally been associated with quality defects in meat products due to acidification, slime formation, and gas production under extended storage conditions [32]. The strains of *L. sakei* (S10) in this study have also been shown to be capable of this. It has been established that their actions are responsible for changes that are associated with spoiling as well as a deterioration in product quality throughout the storage and distribution of sausages.

### 2.2. Growth and Hemolytic Characteristics of Isolated LAB

Table S1 provides a summary of the growth parameters and hemolytic responses of the ten LAB isolates, which are designated as S1–S10. All of the strains showed the ability to grow in the temperature range that was examined, which ranged from 4 °C to 30 °C. This reflects the common settings in which sausage products are stored and distributed. In addition, all of the isolates maintained their viability at NaCl concentrations of 0%, 2%, 4%, and 6%, which is evidence of their remarkable halotolerance and adaptation to salt levels that are typically seen in processed meat products.

On MRS agar that had been supplemented with 5% (*v*/*v*) sheep blood, each strain was streaked and then incubated at 30 °C for 24 h in order to determine its capacity for hemolysis. Molecular metabolites are responsible for the lysis of red blood cells, which is referred to as hemolysis [33]. In this particular experiment, the presence of green discoloration around the colonies is indicative of α-hemolysis, which is caused by partial hemoglobin degradation. On the other hand, β-hemolysis is characterized by a clear zone that indicates full lysis, while γ-hemolysis is characterized by the absence of any visible change in the medium [34]. There was no evidence of erythrocyte degradation in any of the isolates, regardless of whether they exhibited α- or β-hemolytic activity. All of the isolates were classed as γ-hemolytic. Based on these findings, it appears that the strains do not manufacture hemolysins and are not expected to exhibit virulence under the conditions that were examined. Earlier publications have characterized meat-associated LAB as resilient and non-pathogenic bacteria [35,36]. The observed combination of salt and temperature resistance, along with non-hemolytic characteristics in these LAB isolates, is consistent with those earlier reports. These findings provide further evidence that LAB have the potential to be safe for use in applications connected to food, particularly within systems that involve fermented or salt-cured beef products.

### 2.3. Fermentation Profile of Carbohydrate in LAB Isolates

The application of API 50 CH test strips was utilized to evaluate the carbohydrate fermentation profiles of the ten LAB strains (S1–S10). The findings of this evaluation are represented in Table S2. There were distinct consumption patterns that formed among species despite the fact that the majority of isolates were capable of fermenting a wide variety of carbohydrates. The results showed that *L. brevis* (S3) and *W. viridescens* (S8) had the most extensive substrate range, which is indicative of their great metabolic adaptability. In contrast, *L. paracasei* (S2) and *L. plantarum* (S4) exhibited a more restricted capacity for carbohydrate fermentation, which is indicative of a somewhat smaller metabolic capacity.

L-arabinose (4), amygdalin (21), and melezitose (30) were among the specific sugars that were utilized by the four strains of *L. mesenteroides* that were designated as S1, S5, S6, and S9. These strains demonstrated comparable carbohydrate fermentation profiles, with marginal differences in the consumption of individual sugars. Particularly noteworthy is the fact that *L. sakei* (S10) demonstrated robust fermentation activity against typical carbohydrates linked with meat, such as glucose, fructose, and sucrose. The presence of this profile indicates that the organism has adapted physiologically to the high-salt and low-temperature settings that are typical of refined meat products. All of these findings are in agreement with the findings of prior research that identified *L. sakei* as the predominant LAB species in environments that contain fermented meat [37]. Yet, a future study is required to examine the comprehensive safety profile of *L. sakei* (S10) to validate the probiotic’s potential for meat fermentation and storage. There is a high probability that the ecological niches of LAB and their possible technological functions in meat fermentation systems are reflected in the carbohydrate utilization patterns that are particular to each strain.

### 2.4. Synthesis and Characterization of PG-AuNPs

In this study, we employed a green synthesis approach using phloroglucinol (PG), a purified compound derived from the brown seaweed *E. bicyclis*, as the reducing agent to convert ionic gold (Au^3+^) into zerovalent gold nanoparticles (Au^0^). This study retrospectively identifies the bioactive constituents within the *E. bicyclis* extract responsible for reducing activity in the synthesis of EA-AuNPs and EB-AuNPs [38,39]. PG, a well-characterized compound found in *E. bicyclis*, was selected for AuNP synthesis in this study to evaluate its specific efficacy against biofilm-forming LAB.

**Figure 2:**
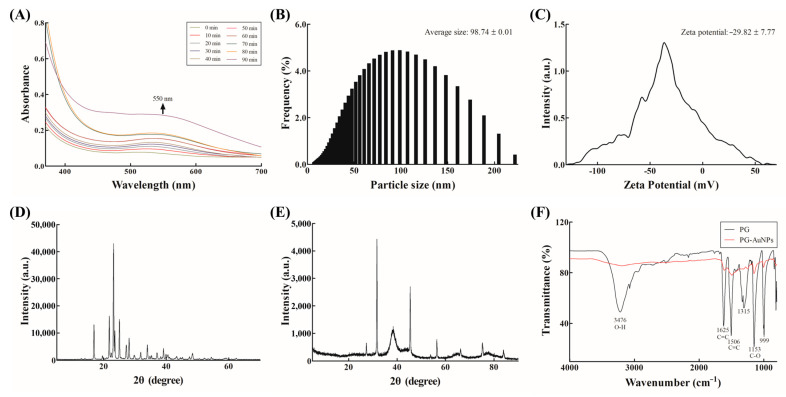
Physicochemical analysis of the PG-AuNPs. (**A**) UV–Vis absorption spectra; (**B**) particle size distribution; (**C**) zeta potential; (**D**) XRD pattern of PG; (**E**) XRD pattern of the PG-AuNPs; and (**F**) FTIR spectra of PG and PG-AuNPs.

PG-AuNPs exhibit localized surface plasmon resonance, typically characterized by strong absorbance in the 500–600 nm range [40]. In this study, successful synthesis of PG-AuNPs was indicated by a visible color change from yellow to a dark red wine color, accompanied by a distinct UV–Vis absorption peak at 550 nm (Figure 2A). DLS analysis determined the average particle size to be 98.74 ± 0.01 nm (Figure 2B). Zeta potential analysis yielded a value of −29.82 ± 7.77 mV, indicating moderate colloidal stability (Figure 2C). This finding is in agreement with prior studies on AuNPs synthesized from marine algae, such as *Sargassum muticum* (−27.9 ± 0.9 mV) [41]. Figure 2D shows the XRD pattern of PG. The XRD patterns of the PG-AuNPs revealed distinct peaks at the 2θ values of 37.80°, 45.30°, 66.08°, and 75.14°, corresponding to the (111), (200), (220), and (311) planes of face-centered cubic gold crystals (Figure 2E) [41]. These crystallographic features are in line with those reported for AuNPs synthesized from various marine algae [42]. FTIR analysis identified functional groups involved in the synthesis and stabilization of the nanoparticles (Figure 2F). A strong band at 3476 cm^−1^ was attributed to phenolic –OH groups, suggesting their role in the reduction of Au^3+^ to elemental gold (Au^0^), consistent with earlier reports [43]. Additional absorption peaks observed in the ranges of 1084–1270 cm^−1^ (C–O stretching) and 1000–1500 cm^−1^ (C=C stretching) confirmed the presence of organic compounds that likely contributed to nanoparticle capping and stabilization [44]. These findings support a mechanism similar to those observed in other natural-extract-based AuNP synthesis [45].

**Figure 3:**
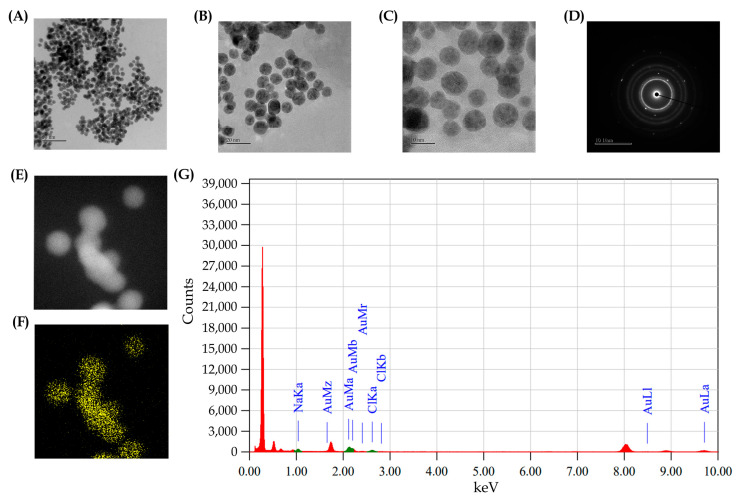
Structural and elemental mapping of the PG-AuNPs using the electron microscopy techniques FE-TEM and EDS. FE-TEM image at a resolution of 50 nm (**A**), 20 nm (**B**), and 10 nm (**C**), and the SAED of the PG-AuNPs (**D**); SEM image of the PG-AuNPs (**E**); mapping of the Au element (**F**); and EDS spectra of the PG-AuNPs (**G**).

FE-TEM further confirmed the spherical morphology of the PG-AuNPs across multiple magnifications (100, 50, 20, and 10 nm) (Figure 3A–D). The formation of spherical NPs is known to be influenced by synthesis parameters such as pH, temperature, and reaction time. Notably, the pH range used in this study (pH 9.0) has been previously associated with the predominance of a spherical nanoparticle morphology [46]. EDS validated the elemental composition and uniform distribution of gold within the synthesized particles (Figure 3E–G). Taken together, the synthesized PG-AuNPs exhibited physicochemical and structural properties consistent with those of other biologically synthesized AuNPs, underscoring their potential applicability in the biomedical and environmental fields.

### 2.5. Assessment of Biofilm Control In Situ Against Pathogens and Biofilm-Forming LAB

Table 1 provides the findings of the in situ studies that were conducted to evaluate the biofilm-inhibitory effects of AuNPs produced from *E. bicyclis*. The effects of the EB, EA, PG, EB-AuNPs, EA-AuNPs, and PG-AuNPs on the prevention of biofilm formation against *P. aeruginosa* and *S. aureus* are presented in Tables S3 and S4, respectively. Taking into consideration the findings of previous research [47], which demonstrated the LAB’s capacity to build biofilms, the S10 strain was chosen as a typical isolate. In addition, the well-known foodborne pathogens *S. aureus* and *P. aeruginosa* were included in the study in order to evaluate the antibacterial activity of the compounds that were evaluated under conditions representative of those actually associated with food.

After being inoculated onto the surface of sausage slices, *P. aeruginosa* displayed significant biofilm formation, with the number of log CFU/g reaching 9.15. There was a considerable reduction in viable cell counts following treatment with *E. bicyclis* extracts, notably EB and EA. The reductions ranged from 1.55 to 2.14 log CFU/g, depending on the concentration of the extracts. The NP formulations, namely the EB-AuNPs and PG-AuNPs, exhibited a discernible level of inhibition. On the other hand, the EA-AuNPs obtained a maximum reduction of 0.44 log CFU/g when administered at a concentration of 1024 μg/mL. Only the PG and PG-AuNPs were successful in lowering viable counts by up to 1.24 log CFU/g. *S. aureus* also developed dense biofilms (9.46 log CFU/g) but displayed selective susceptibility. The PG, EB-AuNPs, and PG-AuNPs were the only substances that were effective. Out of all the LAB strains, S1, S3, and S8 did not exhibit any significant inhibition (the data for these strains are not given). On the other hand, S10 (*L. sakei*), a strain that is well-known for its ability to survive in environments containing meat, showed a significant sensitivity to the EA, PG, and PG-AuNP treatments. The production of biofilms by S10 was decreased by 1.10–1.78 log CFU/g, indicating that S10 has the potential to serve as a model organism for the evaluation of antimicrobial methods that target beneficial yet persistent biofilm-forming LAB. A modest discrepancy in activity between PG and PG-AuNPs in this investigation could be attributed to the composition of the MRS standard media utilized for the LAB strain S10. Previous studies showed that the effectiveness of PG-AuNPs varied in different media, with lower minimum inhibitory concentrations observed in saliva compared to standard and artificial sputum media [48].

These in situ data highlight the limitations of converting laboratory-scale biofilm inhibition into real-world food systems; they are consistent with earlier findings, and they highlight the difficulties involved. Previous research has revealed similar patterns, pointing out that the biofilm inhibition observed in vitro frequently does not translate efficiently in situ due to the complexity and unpredictability of the food matrix [49,50,51]. Due to this constraint, it is of utmost importance to evaluate the effectiveness of antimicrobials under settings that are representative of their actual application in order to guarantee that they are applicable in practice.

Among all tested agents, PG and its nanomaterials PG-AuNP consistently demonstrated the strongest inhibitory activity, particularly against *P. aeruginosa* and *L. sakei* S10. The present study’s findings are in close agreement with those of the previous studies. Coated gold nanoparticles (AuNPs) have been developed to enhance antibiofilm activity against various pathogenic bacteria. AuNPs coated with N-acylated homoserine lactonase proteins showed potent antibiofilm effects against multidrug-resistant *Proteus* species [52]. Cinnamaldehyde-coated AuNPs effectively eradicated biofilms of both Gram-negative and Gram-positive bacteria [23]. Antibiotic-coated AuNPs demonstrated improved antimicrobial properties and biofilm eradication [53]. Furthermore, previous reports showed that the assessed polyphenolic compounds and their nanoformulations show promise in disrupting microbial adhesion and biofilm development on food-contact surfaces [54]. These natural compounds exhibit antibacterial effects through various mechanisms, including membrane disruption and biofilm eradication [54,55]. However, their inherent physicochemical properties limit their effectiveness. Nano formulations offer solutions to overcome these challenges by enhancing bioavailability and controlled release [56]. Thus, due to the inherent antimicrobial and antibiofilm properties of polyphenol, when combined with metallic nanoparticles, it creates synergistic effects, improving antimicrobial efficacy and overcoming limitations such as low solubility and bioavailability [57,58]. These findings lend support to the concept that polyphenolic chemicals and their nanoparticle formulations can effectively inhibit bacterial adherence and biofilm formation on food-contact surfaces. However, in the current study, rather than the PG-AuNPs having a higher efficacy than the PG, there was only a very slight difference in the activity. This suggests that either the surface of the food contact or the composition of the media used for the biofilm assays might have affected this, a factor that needs to be investigated in the future. The observed in situ efficacy indicates that *E. bicyclis* extracts and PG, which were utilized as reducing agents in the synthesis of AuNPs, have significant promise as functional additives for improving microbiological safety and increasing the shelf life of ready-to-eat meat products.

## 3. Limitations of the Present Study

Although *Eisenia bicyclis* extracts and gold nanoparticle (AuNP) formulations showed potential antibiofilm characteristics, this work has certain drawbacks. Surprisingly, the efficacy of these medications in situ was substantially less than in vitro. This is most likely related to the food matrix’s complexity and variety, which may influence antibacterial activity. Furthermore, the study eliminated controls, including chemically synthesized AuNPs, which would have provided a better understanding and comparative study with the green chemistry-based synthesized AuNPs. Furthermore, while *L. sakei* S10 was a typical strain, the study’s breadth was limited to two pathogens and a single LAB model, restricting its ability to be broadly relevant to a diverse microbial community. A detailed safety analysis of these isolates, followed by an in-depth characterization of their biofilm-forming components, has not been conducted, which is another limitation of the studies. Practical implementation issues remain unresolved due to the scarcity of long-term safety studies and sensory evaluations of treated meat products. These drawbacks highlight the need for additional research on a broader range of microbial targets, realistic application settings, and rigorous safety investigations.

## 4. Materials and Methods

### 4.1. Isolation of LAB

**Figure 4:**
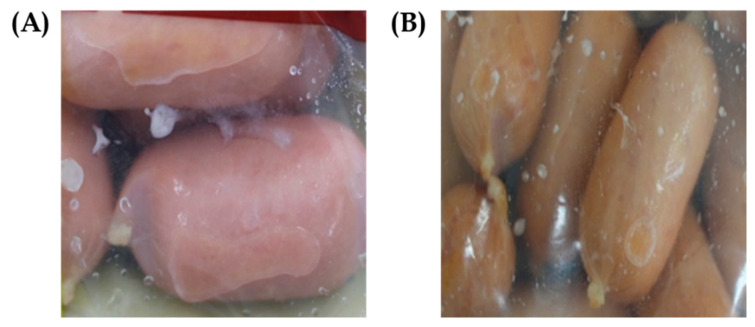
Formation of biofilms by LAB on the surface of pressed ham (**A**) and the surface of Vienna sausages (**B**) during refrigerated storage.

For the purpose of conducting experimental research, Dynesoze Co., Ltd. (Seoul, Republic of Korea) provided processed meat products that had created biofilms. These items were utilized for the analysis shown in Figure 4. Over the course of 2 min, a stomacher manufactured by Interscience in St. Nom, France, was used to homogenize 25 g of each sample in 225 mL of sterile phosphate-buffered saline (PBS) with a concentration of 0.1 mL. MRS agar (Difco, Detroit, MI, USA) was added with 0.002% (*w*/*v*) bromocresol purple (Sigma-Aldrich, St. Louis, MO, USA) after the homogenates were serially diluted in sterile PBS. The plates were then placed on the agar. Following incubation at a temperature of 30 °C for 24 to 48 h, colonies with yellow halos, which indicated the presence of organic acid secretion, were separated and further purified to be used as possible candidates for lactic acid bacteria.

### 4.2. Taxonomic Identification of LAB Isolates

The AccuPrep^®^ Genomic DNA Extraction Kit (Bioneer, Daejeon, Republic of Korea) was utilized in order to extract genomic DNA from the strains that were originally isolated. Universal primers 27F (5′-AGAGTTTGATCMTGGCTCAG-3′) and 1492R (5′-GGTTACCTTGTTACGACTT-3′) were used to amplify the 16S rRNA gene via PCR. The amplified fragments were sequenced by Bionics (Seoul, Republic of Korea), and the obtained sequences were analyzed through the NCBI BLAST tool to determine bacterial identity.

### 4.3. Characterization of Isolated LAB

To characterize the isolated LAB, their growth responses to different temperatures, salt concentrations, and hemolytic properties were evaluated. Growth was assessed at 4 °C, 10 °C, 20 °C, and 30 °C and at NaCl concentrations of 0%, 2%, 4%, and 6%, simulating sausage distribution conditions. For this purpose, the strains were cultured on MRS agar for 24 h. Hemolytic activity was assessed by streaking the isolates onto MRS agar supplemented with 5% (*v*/*v*) sheep blood (KisanBio, Seoul, Republic of Korea) and incubating the plates at 30 °C for 24 h. Hemolytic properties were determined by the presence of clear zones surrounding the colonies.

### 4.4. Biochemical Characterization of LAB Isolates

By utilizing API 50 CH test strips (BioMérieux, Marcy-l’Étoile, France), we were able to examine the carbohydrate utilization profiles as well as the biochemical features of the LAB isolates. A 2.0 McFarland turbidity standard was used to prepare bacterial suspensions, which were then dispensed into the wells of the test strip. The suspensions were prepared in API 50 CHL medium. Sterile mineral oil was layered over each well in order to produce anaerobic conditions. After the strips had been injected, they were incubated at a temperature of 30 °C for 48 h. The transformation of the hue from violet to yellow was indicative of the fermentation of carbohydrates, whereas the formation of a black coloring was associated with the hydrolysis of esculin.

### 4.5. Preparation and Characterization of AuNPs

The synthesis of AuNPs was carried out by employing PG (Sigma-Aldrich, St. Louis, MO, USA) as a reducing agent in accordance with a modified approach that was reported by Oh et al. [38]. While gold (III) chloride trihydrate (Sigma-Aldrich) was prepared at a concentration of 1 mM, and its pH adjusted to 9.0 using sodium hydroxide, PG was dissolved in distilled water to a final concentration of 2% (*w*/*v*). The PG solution was added drop by drop to the gold chloride solution until a characteristic dark red wine color formed. This showed that the creation of AuNPs had occurred. Utilizing a spectrophotometer (Synergy HTX; Biotek, Winooski, VT, USA), the synthesis process was tracked by recording the UV–Vis absorption spectra (200–700 nm) at 10-minute intervals.

Particle size, stability, structure, and morphology were all evaluated in relation to the PG-AuNPs that were manufactured. Through the utilization of dynamic light scattering (DLS; Litesizer 500, Anton Paar GmbH, Graz, Austria), the size and zeta potential of the AuNPs were determined. In order to confirm the conversion of Au^3+^ to Au^0^, Fourier transform infrared spectroscopy (FTIR; FT-4100, JASCO, Tokyo, Japan) was utilized to identify functional groups involved in the reduction process. In particular, phenolic –OH groups were confirmed. X-ray diffraction (XRD; Ultima IV, Rigaku, Tokyo, Japan) revealed characteristic peaks at the 2θ values of 37.80°, 45.30°, 66.08°, and 75.14°, which corresponded to the crystalline structure of gold nanoparticles. While energy-dispersive X-ray spectroscopy (EDS) analysis proved the elemental composition and uniform distribution of gold throughout the sample, field-emission transmission electron microscopy (FE-TEM; JEM-F200, JEOL, Tokyo, Japan) confirmed the spherical morphology of the nanoparticles. FE-TEM was applied to the sample.

### 4.6. Biofilm-Inhibitory Activity of LAB Isolates

The in situ experiment was conducted under in vitro conditions using concentrations previously reported to inhibit biofilm formation [38]. To evaluate antibiofilm activity, PG—a key compound derived from *E. bicyclis*—was tested alongside two crude extracts (EB and EA). Their corresponding AuNP formulations (PG-AuNPs, EA-AuNPs, and EB-AuNPs) were also evaluated in vitro at the same concentrations shown to exert inhibitory effects in prior studies [47]. The sub-MIC concentrations of PG-AuNPs, EA-AuNPs, and EB-AuNPs were used for evaluating the biofilm-inhibitory effects towards the *P. aeruginosa*, *S. aureus*, and *L. sakei* S10 (Table 1, Tables S3 and S4).

Although *L. sakei* and other LAB strains play important roles in the microbial ecology of fermented meat products, biofilm-forming pathogenic bacteria persist in food manufacturing environments, posing a substantial issue. To comprehensively evaluate the biofilm-inhibitory effects of *E. bicyclis*-derived AuNPs, this study included not only the isolated LAB strains but also representative spoilage and foodborne pathogens, specifically *P. aeruginosa* and *S. aureus*, as target organisms. The bacterial pathogens *P. aeruginosa* (KCTC 1637) and *S. aureus* (KCTC 1916) were obtained from the Korean Collection for Type Cultures (KCTC, Daejeon, Republic of Korea). Both of these species are considered to be pathogenic. These potentially harmful bacteria were grown in growth media, such as tryptic soy broth (TSB), which was employed for the cultivation process.

Commercial sausages were purchased, stored at −80 °C, and used for experimental analysis. The sausages were cut into 2 × 2 cm pieces using a sterile knife and subjected to UV-C irradiation for 30 min to eliminate background microbial contamination [59]. Cultured LAB and pathogenic strains were serially diluted to concentrations of 10^4^–10^5^ CFU/mL. The UV-C-treated sausage pieces were immersed in the diluted bacterial suspensions for 2 min, air-dried for 20 min, and subsequently incubated at 30 °C for 24 h to achieve a final inoculation level of 10^2^–10^3^ CFU/g [60]. Sausage pieces were incubated and then homogenized in 0.1 M PBS for two minutes using a stomacher after a 10-fold dilution. After the 10-fold serial dilution, 100 µL of the samples were spread out and plated on an MRS agar plate for the growth of LAB cells and on a TSA plate for the growth of *P. aeruginosa* and *S. aureus* cells. Plates were incubated at 30 °C for 24 h for LAB and at 37 °C for pathogens. The enumeration of colony-forming units was accomplished by counting several separate colonies.

### 4.7. Statistical Analysis

Every experiment was carried out three times in a separate and independent manner. We used GraphPad Prism version 7.0 (GraphPad Software Inc., San Diego, CA, USA) to perform the analysis of the data, and we used a one-way analysis of variance to determine the statistical significance of the findings.

## 5. Conclusions

In this work, ten different strains of LAB were isolated from meat products, and their physiological and biochemical features were evaluated. These properties included growth behavior, hemolytic activity, and carbohydrate utilization. As a result of its remarkable biofilm-forming potential and tolerance to environmental circumstances, *L. sakei* S10 has been identified as an appropriate model strain for the purpose of assessing antimicrobial methods in the food matrix.

It was established that the in situ administration of *E. bicyclis* extracts, PG, and their respective AuNP formulations inhibited biofilms by strain specificity. It is important to note that the PG and PG-AuNPs consistently demonstrated the most potent inhibitory effects, particularly against *P. aeruginosa* and *L. sakei* S10. It is essential to evaluate the effectiveness of antimicrobial agents under conditions that are more representative of real-world applications, despite the fact that a number of compounds were found to be effective in laboratory settings. However, their biofilm-suppressing activities were significantly diminished in the food matrix.

These findings indicate that polyphenolic compounds—such as PG, especially in nano-formulated forms—have the potential to be incorporated into food packaging materials or surface coatings to improve the microbiological safety and quality of ready-to-eat meat products.

## Figures and Tables

**Table 1:** Effects of *Eisenia bicyclis* extracts and AuNPs against *Lactobacillus sakei* S10 biofilms on the sausage surface.

Samples	Treated Concentration (μg/mL)	Cell Growth of the Biofilm (Log CFU/g)
S10 strain (control)	-	6.38 ± 0.01
EA-AuNPs	1024	5.23 ± 0.11
PG	1024	4.60 ± 0.60
PG-AuNPs	1024	5.00 ± 0.00

## Data Availability

Available upon request.

## Supplementary Materials

The following supporting information can be downloaded at: https://www.mdpi.com/article/10.3390/antibiotics14070637/s1. Table S1. Growth characteristics at different temperatures and NaCl concentrations, and hemolytic activity of LAB isolates. Table S2. API 50 CH fermentation patterns of isolated LAB strains. Table S3. Effects of *Eisenia bicyclis* extracts and AuNPs against *Pseudomonas aeruginosa* biofilms on sausage surface. Table S4. Effects of *Eisenia bicyclis* extracts and AuNPs against *Staphylococcus aureus* biofilms on sausage surface.
