# Supplementary material for: Biofilm-Forming Lactic Acid Bacteria in Sausages: Isolation, Characterization, and Inhibition Using Eisenia bicyclis-Based Nanoparticles
Source: Antibiotics (Basel). 2025 Jun 22;14(7):637. doi: 10.3390/antibiotics14070637 (PMC12291735; doi:10.3390/antibiotics14070637)
Supplement: Supplementary file 1 [file antibiotics-14-00637-s001.zip › antibiotics-3692255-supplementary.pdf]

Table S1. Growth characteristics at different temperatures and NaCl concentrations, and hemolytic activity of LAB isolates.

No.	Strain	Temperature (°C)				NaCl Concentration (%)				Hemolysis Type
		4	10	20	30	0	2	4	6	
S1	*Leuconostoc mesenteroides*	+	+	+	+	+	+	+	+	γ (non-hemolytic)
S2	*Lacticaseibacillus paracasei*	+	+	+	+	+	+	+	+	γ (non-hemolytic)
S3	*Lactobacillus brevis*	+	+	+	+	+	+	+	+	γ (non-hemolytic)
S4	*Lactiplantibacillus plantarum*	+	+	+	+	+	+	+	+	γ (non-hemolytic)
S5	*Leuconostoc mesenteroides*	+	+	+	+	+	+	+	+	γ (non-hemolytic)
S6	*Leuconostoc mesenteroides*	+	+	+	+	+	+	+	+	γ (non-hemolytic)
S7	*Lactobacillus brevis*	+	+	+	+	+	+	+	+	γ (non-hemolytic)
S8	*Leuconostoc citreum*	+	+	+	+	+	+	+	+	γ (non-hemolytic)
S9	*Weissella viridescens*	+	+	+	+	+	+	+	+	γ (non-hemolytic)
S10	*Latilactobacillus sakei*	+	+	+	+	+	+	+	+	γ (non-hemolytic)

+ indicates visible growth under the respective conditions

Table S2. API 50 CH fermentation patterns of isolated LAB strains

	S1	S2	S3	S4	S5	S6	S7	S8	S9	S10
Glycerol	+	+	+	+	+	+	+	+	+	+
Erythritol	+	+	+	+	+	+	+	+	+	+
D-Arabinose	+	+	+	+	+	+	+	+	+	+
L-Abarbinose	+	+	+	+	+	+	+	+	+	+
D-Ribose	-	+	-	-	-	+	-	+	-	-
D-Xylose	-	-	-	-	-	-	+	+	-	-
L-Xylose	-	+	-	+	-	-	+	+	-	+
D-Adonitol	+	+	+	+	+	+	+	+	+	+
Methyl-β-D-Xylpyranoside	+	-	+	+	+	+	+	+	+	+
D-Galactose	+	+	+	+	+	+	+	+	+	+
D-Glucose	+	-	+	-	-	-	+	+	+	-
D-Fructose	-	-	+	-	-	-	-	-	-	-
D-Mannose	-	-	-	-	-	-	-	-	-	-
L-Sorbose	-	-	+	-	-	-	-	-	-	-
L-Rhamnose	+	-	+	+	+	+	+	+	+	+
Dulcitol	+	+	+	+	+	+	+	+	+	+
Inositol	+	+	+	+	+	+	+	+	+	+
D-Mannitol	+	+	+	+	+	+	+	+	+	+
D-Sorbitol	+	-	+	-	+	+	+	+	+	+
Methyl-α-D-Mannopyranoside	+	-	+	-	+	+	+	+	+	+
Methyl-α-D-Glucopyranoside	+	+	+	+	+	+	+	+	+	+
N-Acetylglucosamine	-	+	+	+	-	-	+	+	-	+
Amygdalin	-	-	+	-	-	-	-	-	-	-
Arbutin	+	-	+	-	+	+	+	+	+	+
Esculin ferric citrate	+	-	+	-	+	+	-	+	+	+
Salicin	+	+	+	+	+	+	+	+	+	+
D-Cellobiose	+	-	+	-	+	+	-	+	+	+
D-Maltose	+	-	+	-	+	-	+	+	+	+
D-Lactose	-	-	-	-	-	-	-	-	-	-
D-Melibiose	+	-	+	-	+	+	+	+	+	+
D-Saccharose	-	+	+	-	-	-	+	+	-	-
D-Trehalose	-	-	+	-	-	-	-	-	-	-
Inulin	-	-	+	-	-	-	-	-	-	-
D-Melezitose	+	-	+	+	+	+	+	+	+	+
D-Raffinose	+	-	+	-	+	+	+	+	+	+
Amidon	-	+	+	-	+	-	+	+	-	+
Glycogen	+	+	+	+	+	+	+	+	+	+
Xylitol	+	+	+	+	+	+	+	+	+	+
Gentiobiose	+	+	+	+	+	+	+	+	+	+
D-Turanose	+	+	+	-	+	-	+	+	+	-
D-Lyxose	-	+	+	-	-	-	-	+	-	+
D-Tagatose	+	+	+	+	+	+	+	+	+	+
D-Fucose	+	-	+	+	+	+	+	+	+	+
L-Fucose	+	+	+	+	+	+	+	+	+	+
D-Arabitol	+	+	+	+	+	+	+	+	+	+
L-Arabitol	+	+	+	+	+	+	+	+	+	+
Potassium Gluconate	+	+	+	+	+	+	+	+	+	+
Potassium 2-Ketogluconate	+	-	-	-	+	+	-	-	-	-
Potassium 5-Ketogluconate	+	+	+	+	+	+	+	+	+	+

Table S3. Effects of *Eisenia bicyclis* extracts and AuNPs against *Pseudomonas aeruginosa* biofilms on sausage surface.

Samples	Treated concentration (μg/mL)	Cell growth of the biofilm (Log CFU/g)
P. aeruginosa (Control)	-	9.15±0.06
EB	2,048	7.36±0.13
	1,024	7.59±0.33
EA	1,024	7.56±0.46
	512	8.92±0.22
PG	2,048	6.81±0.04
EB-AuNPs	2,048	7.65±0.16
	1,024	8.05±1.26
	512	8.29±0.60
EA-AuNPs	1,024	8.71±0.25
	512	9.22±0.02
PG-AuNPs	2,048	7.78±0.43
	1,024	8.00±0.48
	512	8.15±0.61

Table S4. Effects of *Eisenia bicyclis* extracts and AuNPs against *Staphylococcus aureus* biofilms on sausage surface.

**Samples**	**Treated concentration (μg/mL)**	**Cell growth of the biofilm (Log CFU/g)**
S. aureus (Control)	-	9.46±0.01
EB	512	9.04±0.10
EA	128	9.23±0.20
PG	2,048	8.86±0.11
	1,024	8.35±0.54
EB-AuNPs	512	9.32±0.15
	256	9.13±0.03
	128	9.08±0.02
	64	9.19±0.02
EA-AuNPs	256	9.45±0.02
	128	9.57±0.17
PG-AuNPs	2,048	8.22±0.10
	1,024	9.38±0.07
